# Monitoring the progression of metastatic breast cancer on nanoporous silica chips

**DOI:** 10.1098/rsta.2011.0444

**Published:** 2012-05-28

**Authors:** Jia Fan, Xiaoyong Deng, James W. Gallagher, Haiyu Huang, Yi Huang, Jianguo Wen, Mauro Ferrari, Haifa Shen, Ye Hu

**Affiliations:** 1 Department of Nanomedicine, The Methodist Hospital Research Institute, Houston, TX 77030, USA; 2 CAS Key Laboratory for Biological Effects of Nanomaterials and Nanosafety, National Center for Nanoscience and Technology, Beijing 100190, People's Republic of China; 3 Institute of Nanochemistry and Nanobiology, School of Environmental and Chemical Engineering, Shanghai University, Shanghai 200444, People's Republic of China; 4 Department of Pathology, The Methodist Hospital Research Institute, Houston, TX 77030, USA

**Keywords:** nanoporous silica, breast cancer lung metastasis, low molecular weight protein, proteomic biomarker, early diagnosis

## Abstract

Breast cancer accounted for 15 per cent of total cancer deaths in female patients in 2010. Although significant progress has been made in treating early-stage breast cancer patients, there is still no effective therapy targeting late-stage metastatic breast cancers except for the conventional chemotherapy interventions. Until effective therapy for later-stage cancers emerges, the identification of biomarkers for the early detection of tumour metastasis continues to hold the key to successful management of breast cancer therapy. Our study concentrated on the low molecular weight (LMW) region of the serum protein and the information it contains for identifying biomarkers that could reflect the ongoing physiological state of all tissues. Owing to technical difficulties in harvesting LMW species, studying these proteins/peptides has been challenging until now. In our study, we have recently developed nanoporous chip-based technologies to separate small proteins/peptides from the large proteins in serum. We used nanoporous silica chips, with a highly periodic nanostructure and uniform pore size distribution, to isolate LMW proteins and peptides from the serum of nude mice with MDA-MB-231 human breast cancer lung metastasis. By matrix-assisted laser desorption/ionization time-of-flight mass spectrometry and biostatistical analysis, we were able to identify protein signatures unique to different stages of cancer development. The approach and results reported in this study possess a significant potential for the discovery of proteomic biomarkers that may significantly enhance personalized medicine targeted at metastatic breast cancer.

## 1. Introduction

Breast cancer is the most frequently occurring malignancy and the second most frequent cause of cancer death in women in the USA and other Western countries [1]. Significant progress has been made on overall survival rates in breast cancer. The 5 year overall survival rate of women diagnosed with stage I–III breast cancer has been steadily improving for decades [2,3]. These improvements in survival have primarily been attributed to the availability of modern diagnostic tools that allow for early detection [4,5], and the increased use of adjuvant systemic therapies [6,7]. However, improved states related to breast cancer metastasis have been discouraging, with only marginal improvement in some reports [2], to no marked improvement reported in other studies [3] in the survival rate of relapsed metastatic patients. The identification of biomarkers for the detection of cancer metastasis in the early stage may hold the key to the management of metastatic breast cancer.

The process of metastasis can be divided into several steps, including the detachment of tumour cells from the primary tumour, invasion, migration, intravasation, survival in the vasculature, extravasation and colonization of the secondary site [8]. There are also reports of circulating tumour cells that might be responsible for tumour metastasis [9,10]. During the steps of metastasis, the cancer tissue sheds cells and/or debris into the blood stream. Each cell leaves behind proteins and peptides in the blood, either as waste or as signals to neighbouring cells. These residual proteins and peptides may reveal new insights into the evolution of the metastatic process.

If isolated and identified, proteomic biomarkers have the potential to revolutionize the practice of medicine by providing the capability to diagnose disease at a pre-symptomatic stage, closely tracking responses to therapy, in addition to representing an important enabler to personalized medicine [11,12]. While there is value in the ability to identify individual proteins that may act as single-disease biomarkers, many diseases will more often be characterized by complicated ‘fingerprints’ involving a number of proteins, peptides and fragments [13]. Although high molecular weight (HMW) proteins in blood have been extensively studied, little is known about the composition of the low molecular weight (LMW) proteins and peptides owing to the difficulties in isolation and purification of this population. These substances may circulate at very low levels and are difficult to detect in the presence of the abundance of proteins such as albumin [14].

Biomolecules can now be identified via mass spectrometry (MS), yielding a spectrum of the quantity of materials as a function of their molecule (and molecular fragment) mass [15]. The ability to quantify all biomolecules, from very large proteins down to peptides that comprise protein/peptide fragments as low as a thousand daltons, is now emerging. Several technologies for sample fractionation prior to MS analysis have been developed to address these limitations, including conventional two-dimensional gel electrophoresis and beads equalization. In spite of these advances, however, the detection of low abundance markers and small proteins has remained a critical challenge owing to the labour-intensive and low-throughput procedures, which have offered limited suitability for clinical applications [16–18].

In our study, we employed a mouse model of lung metastasis with the human breast cancer cell line MDA-MB-231, which is a triple negative cell line lacking expression of the oestrogen receptor (ER), the progesterone receptor (PR) and the Her2 receptor (HER2). The lung is one of the most frequent sites of breast cancer metastasis. Approximately, 60 per cent of breast cancer patients have had cancer cells eventually spread to their lungs. In 21 per cent of all cases, the lung is the only metastatic organ [19]. Triple negative breast cancer primarily affects younger women (atypical for breast cancer in general), women with African–American descent and women carrying a BRCA1 mutation [20]. Its aggressive nature and high rate of reoccurrence, as well as this cancer's resistance to common targeted therapies, are attributed to the lack of ER, PR and HER2. Patients in this subgroup must revert to conventional cytotoxic chemotherapy, such as anthracycline and taxane, and endure the substantial burden of adverse effects. While conventional chemotherapy and radiation therapy are initially effective in controlling breast cancer growth, patients frequently relapse over time. We have developed nanoporous silica chips to capture LMW serum peptides and proteins [21–26]. In our study, we used nanoporous silica chips to isolate LMW proteins and peptides from the serum of MDA-MB-231 lung metastatic cancer mice. Matrix-assisted laser desorption/ionization time-of-flight mass spectrometry (MALDI-TOF MS) was used in profiling the serum samples in three distinct groups (control, early stage and late stage). All data were then subjected to principal component analysis (PCA) and vertical scatter plots were drawn to identify signatures unique to the different stages of cancer development.

## 2. Experimental

### (a) Fabrication of nanoporous silica thin films

A typical preparation of the coating sol is as follows. Fourteen millilitres of tetraethyl orthosilicate (TEOS; Sigma-Aldrich, St. Louis, MO, USA) was dissolved in a mixture of 17 ml of ethanol, 6.5 ml of distilled water and 0.5 ml of 2 M HCl and stirred for 1 h at 80^°^C to form a clear silicate solution. Separately, 1.8 g of Pluronic F-127 (BASF Co.) was dissolved in 10 ml of ethanol by stirring at room temperature. The coating solution was prepared by mixing the silicate solution into the triblock copolymer solution followed by stirring of the resulting solution for 2 h at room temperature. The pH of the mixture solution remained around 1.5. The coating sol was deposited on a Si (100) wafer by spin-coating at the spin rate of 2000 r.p.m. for 20 s. To increase the degree of polymerization of the silica framework in the films and to further improve their thermal stability, the films were heated at 80^°^C for 12 h. They were then calcinated at 425^°^C to remove the organic surfactant. The temperature was raised at a rate of 1^°^C min^−1^, and the furnace was heated at 425^°^C for 5 h.

### (b) Characterization techniques

We used several characterization techniques to study the spin-coated, nanoporous silica thin films. By employing a variable-angle spectroscopic ellipsometer (J. A. Woollam Co., M-2000DI) and modelling with CompleteEASE software, the thickness of the thin films and their porosities were measured in the Cauchy model and effective medium approximation (EMA) model, respectively. Ellipsometric optical quantities, the phase (*Δ*) and amplitude (*ψ*), were determined by acquiring spectra at 60^°^, 65^°^ and 70^°^ incidence angles using wavelengths from 300 to 1800 nm. In the Cauchy model, the top layer's thickness, reflective index and model-fitted, parameters *A*_*n*_, *B*_*n*_ and *C*_*n*_ were determined by comparing the experimental data with the model and minimizing the mean square error (usually less than 10). Using the EMA model, the films' porosities were calculated by assuming a certain volume of voids in the pure silica and setting the top layer's thickness obtained by the Cauchy model as a constant. X-ray diffraction (XRD) patterns were obtained on a Philips X'Pert-MPD system with Cu K*α* radiation (45 kV, 40 mA). Scans of *θ*–2*θ* were recorded from all spin-coated films at 1 s and 0.001^°^ steps over the angle range from 0.2^°^ to 6^°^. Transmission electron microscopy (TEM) and scanning transmission electron microscopy (STEM) were used to acquire micrographs of the plane view of nanoporous silica thin films with an FEI Technai (FEI Co.) at a high tension of 200 kV. Ellipsometric porosimetry (Semilab Semiconductor Physics Laboratory Co. Ltd.) was applied to measure the adsorption/desorption isotherm and pore size distribution. Nanopore size distributions were calculated from the desorption branch of the isotherms using the Barrett–Joyner–Halenda (BJH) method. A Nicolet Magna 560 spectrometer was used in the transmission mode for Fourier transform infrared (FTIR) measurements, with wavenumber resolution at 4 cm^−1^, 256 scans and the wavenumber range approximately 4000–520 cm^−1^. During measurements, the FTIR chamber was purged with N_2_ gas to remove water vapour and carbon dioxide.

### (c) Construction of bioluminescent breast cancer cell line

The oestrogen-independent human breast cancer cell line MDA-MB-231 was purchased from the American Type Culture Collection (ATCC), and cultured in high-glucose Dulbecco's modified Eagle's Minimal Essential Medium (DMEM; Invitrogen, Grand Island, NY, USA), which contains 10 per cent foetal bovine serum (FBS; Atlanta Biologicals, Lawrenceville, GA, USA) and 1 per cent penicillin–streptomycin solution (Life Technologies, Inc., Grand Island, NY, USA), in a humidified atmosphere of 5 per cent CO_2_ in air at 37^°^C. The process of constructing the bioluminescent cell line has been published before [27]. Briefly, the firefly luciferase gene (luc2; Promega, Madison, WI, USA) was first cloned into the pcDNA6.2-GFP-DEST vector (Invitrogen) to obtain the fused luciferase/GFP cassette. After purification, the cassette was cloned to a lentivirus backbone (Invitrogen) to form an engineered plasmid and then packaged into virus particles. After transfection with these virus particles, GFP-positive MDA-MB-231 cells were selected by fluorescence-activated cell sorting. Cells were maintained in complete DMEM with 6 μg ml^−1^ blasticidin.

### (d) Animal model

All animal work was done in accordance with a protocol approved by the Institutional Animal Care and Use Committee (IACUC) of The Methodist Hospital Research Institute in Houston, TX, USA. Eight-week-old female nude mice were purchased from Charles River Laboratories (Boston, MA, USA). They were housed in a specific pathogen-free facility under a 12 h light–12 h dark cycle, and fed with a pathogen-free diet and water *ad libitum*. To constitute a mouse model of breast cancer lung metastasis (BCLM), 3×10^5^ MDA-MB-231/luciferase cells were washed and suspended in phosphate buffer solution (PBS), and subsequently injected into the lateral tail vein in a volume of 100 μl. The mice were imaged weekly after tumour cell inoculation. Prior to imaging, the mice were first anaesthetized with isoflorane. They were then treated by intraperitoneal injection of d-luciferin (150 mg kg^−1^ body weight in PBS; Caliper Lifesciences, Hopkinton, MA, USA), and placed onto a warmed stage inside the light-tight chamber of an IVIS Imaging System 200 series (Xenogen, Alameda, CA, USA). In order to collect consistent and the strongest possible bioluminescence, all images were captured by the IVIS system 10 min after luciferin. A semiquantitative region of interest (ROI) analysis was performed with the Live Image v. 4.2 software, and the intensity of bioluminescence was used as the parameter to measure growth and to rank the stages of lung metastasis.

### (e) On-chip sample fractionation

After collecting whole blood samples from the mice, the samples were allowed to clot at room temperature in microcentrifuge tubes (Eppendorf, USA) for 1 h. The samples were centrifuged twice at 3000 r.p.m. for 15 min. The separated serum was then removed with a pipette and placed in aliquots of 40 ml in new microcentrifuge tubes. The serum samples were stored at −80^°^C until used, while the remaining components of the mouse blood were discarded. Pretreatment of the samples began with thawing the serum samples on ice. A pretreatment solution of 50 per cent acetonitrile (ACN; Sigma-Aldrich, St. Louis, MO, USA) and 0.1 per cent trifluoroacetic acid (TFA; Sigma-Aldrich, St. Louis, MO, USA) in deionized water was prepared and vortex-mixed. This pretreatment solution was added to the thawed serum samples in a 1:9 ratio to obtain final concentrations of ACN and TFA of 5 and 0.01 per cent, respectively. The samples were then mixed on a table vortexer at room temperature for 30 min.

After mixing, the samples were again spun in a centrifuge, this time at 5000 r.p.m. for 5 min. Meanwhile, the porous silica chips were first baked in an oven at 160^°^C overnight. Compressed air was used to remove any dust from the surface. Chambered coverslips (Sigma-Aldrich, St. Louis, MO, USA) were washed with 100 per cent ethanol and cut to size. They were placed on the chip surface and secured using forceps to ensure complete adherence to the chip. Five microlitres of each sample were pipetted into each well, ensuring that no precipitate in the base of a tube was placed on the chip. The samples were allowed to incubate at room temperature in a 100 per cent humidified chamber for 30 min. The serum samples were pipetted up from the well and discarded. Each well was washed four times with 10 μl of deionized water. The elution buffer was the same as the pretreatment solution (50% ACN and 0.1% TFA in deionized water). Five microlitres of elution buffer were applied to each well and mixed by pipette, up and down 30 times, while rotating the pipette tip around in a random manner. Finally, the elution buffers were pipetted up and placed in new microcentrifuge tubes. These tubes were then stored at −80^°^C until ready for MALDI-TOF analysis.

### (f) Matrix-assisted laser desorption/ionization time-of-flight analysis

The samples from the chips were removed from the freezer and allowed to thaw on ice. Then 0.5 μl of each sample was spotted on the MALDI target plate and allowed to dry completely; and 0.5 μl of the matrix solution was spotted on the target plate and allowed to dry. We used a matrix solution of 5 g l^−1^ of α-cyano-4-hydroxycinnamic acid (CHCA) in 50 per cent ACN and 0.1 per cent TFA. Mass spectra were acquired using the Applied Biosystems 4700 MALDI TOF/TOF Analyzer (Applied Biosystems, Inc., Framingham, MA, USA) at the M.D. Anderson Cancer Center, Houston, TX, USA. Samples were analysed using positive reflector mode (laser intensity 4200, 3000 shots/sample and mass range 800–5000 Da with target mass of 2000 Da).

### (g) Statistical analysis

The resultant MALDI-TOF data were processed using ConvertPeaklist software to generate text files, listed in mass–intensity pairs. These text files were then imported into SpecAlign software for visualization and average spectrum generation. The PCA and *t*-test analysis were performed by MarkerView software v. 1.2.1 (AB SCIEX, Concord, Canada). Six repeated spectra for each sample were selected and imported into MarkerView. The data were normalized to total area sum. PCA processing was carried out with a Pareto scaling. The *t*-value and corresponding *p*-value were also calculated using MarkerView between BCLM and the control groups.

## 3. Results and discussion

**Figure 1.**
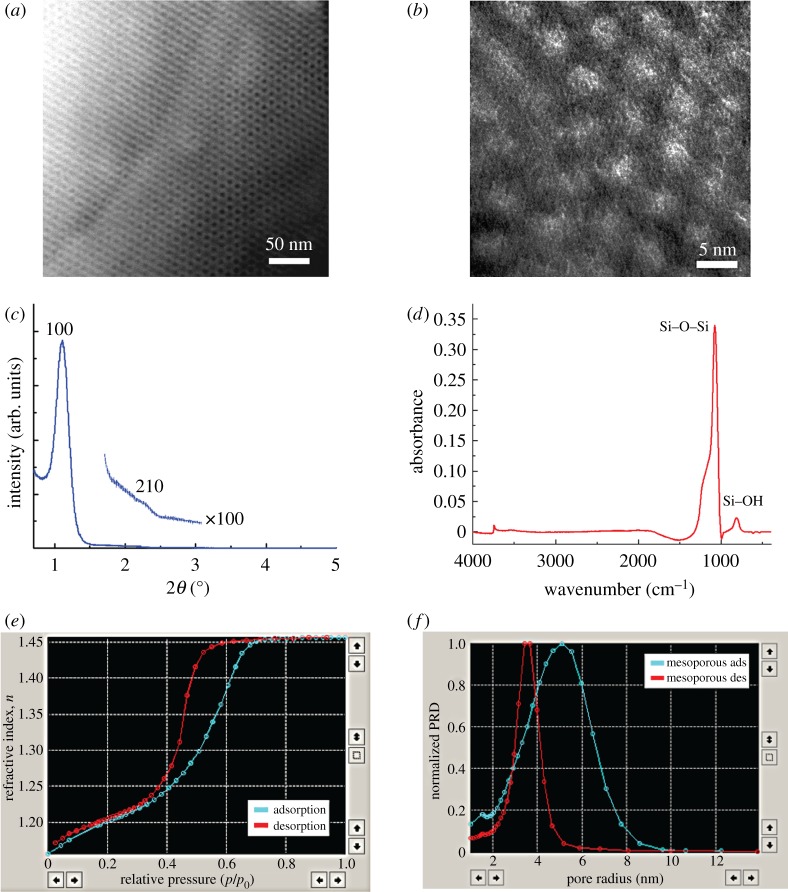
The physical characterization of nanoporous silica thin films: (*a*) STEM plane view of nanoporous silica film; (*b*) TEM plane view; (*c*) XRD pattern; (*d*) FTIR spectrum; (*e*) adsorption/desorption isotherm; and (*f*) pore size distribution. (Online version in colour.)

With the specified molar ratio (1:0.006) of silicate to F-127 copolymer designed in this study, the final product yielded a three-dimensional honeycomb-like nanostructure hexagonally arranged on the substrate, as depicted in its STEM plane view at low magnification (figure 1*a*) and its TEM plane view at high magnification (figure 1*b*). The XRD plot (figure 1*c*) represents the pattern of a thin film, also confirmed as a hexagonal nanostructure with the appearance of a high-intensity (100) reflection peak at 2*θ*=1.026^°^ (*d*_100_=8.604 nm) and a lower-intensity peak at (210) with *d*_210_=4.600 nm. After high-temperature calcination treatment, an oxygen plasma was applied to restore the defective points (Si–H) on the nanoporous silica surface and generated high-density Si–OH groups as shown by the FTIR pattern (figure 1*d*), providing bonding configuration characterization and the molecular structure of the specific groups within the porous framework. The peak at 793 cm^−1^ represents various molecular species, including Si–OH, Si–OH–OH_2_ and Si–CH–O. In addition, the peak at 1074 cm^−1^ indicates that the silica networks comprise Si–O–Si backbones. The FTIR results indicate that the silica surface affinity constitutes an advantage in the enrichment of LMW proteins. The adsorption/desorption isotherms (figure 1*e*) signal an H1 hysteresis loop attributed to cylindrically shaped pores (sloping adsorption branch and nearly vertical desorption branch), indicating a nanoporous structure with opened interconnecting channels. As depicted in figure 1*f*, the pore size distributions on the films display a symmetric and sharp single peak, at a pore diameter of 3.72 nm from the adsorption curve, which strongly points to a silica framework with uniform nanotexture distribution.

**Figure 2.**
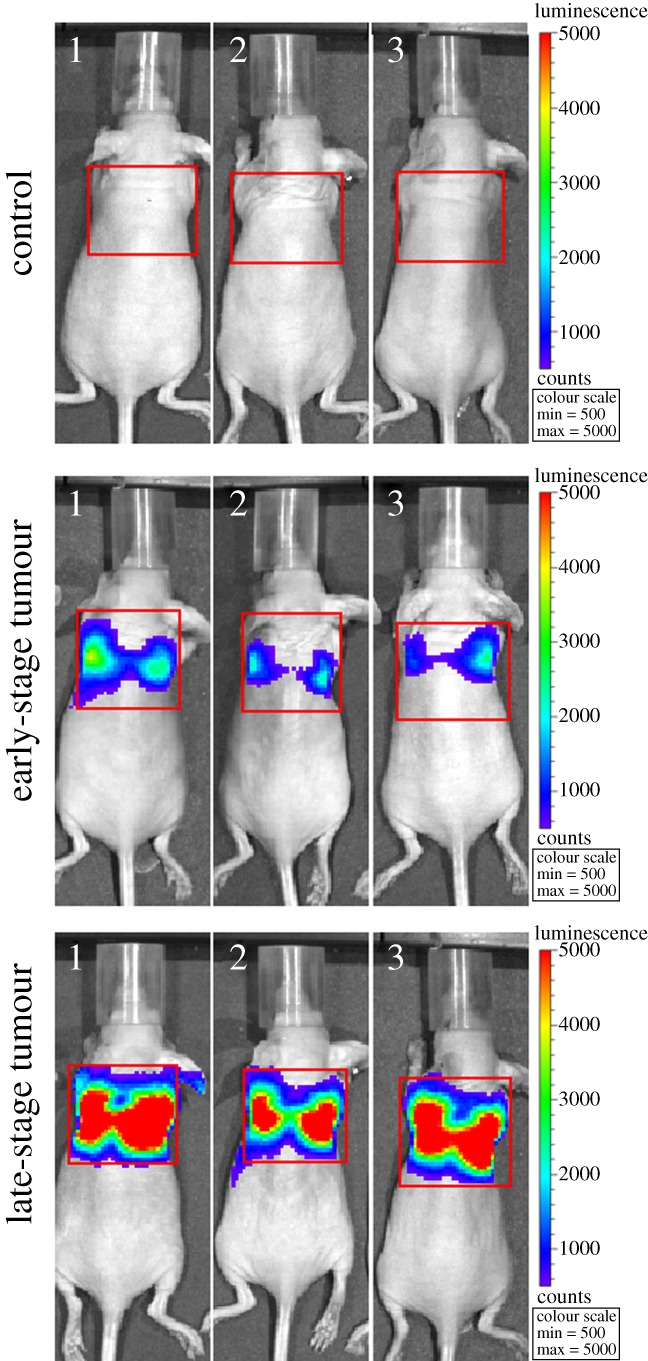
Imaging of MDA-MB-231 tumour growth in nude mice. The MDA-MB-231 human breast cancer cells were engineered with a luciferase gene. Tumour growth was monitored by bioluminescent intensity with a Xenogen IVIS 200 imaging system. The areas of lung tumour are shown boxed, and quantitation of bioluminescent intensity is summarized in table 1. (Online version in colour.)

We generated mouse models of BCLM with MDA-MB-231 human breast cancer cells. These cancer cells were engineered with an overexpression of the luciferase gene. Tumour growth was monitored in live animals by the increase in bioluminescence after injection of d-luciferin into the intraperitoneal cavity. The luciferase enzyme in cancer cells digested d-luciferase, and turned this otherwise non-fluorescent substrate into a strongly fluorescent dye. After inoculation with 3 million cells per mouse, we measured an increase of bioluminescence throughout the next 8–10 weeks (figure 2). Most mice would die of tumour growth in the lung after 10 weeks. We arbitrarily divided the tumour mice into early-stage breast cancer lung metastasis (BCLM-ES) tumour and late-stage breast cancer lung metastasis (BCLM-LS) tumour groups, based on bioluminescent intensity (figure 2), and collected blood by retro-orbital bleeding for proteomic analysis to identify biomarkers that reflect changes in tumour progression. Samples from the BCLM-ES mice were collected two to three weeks post-tumour inoculation, and those from the BCLM-LS tumour mice were taken four weeks later. The growth of metastatic tumours can be judged by the six- to ninefold increase of bioluminescent intensity between the early-stage and late-stage tumours (table 1). We also collected blood samples from normal nude mice as negative controls. There were eight animals per group in this experiment.

**Table 1.** Bioluminescent intensity of tumours in nude mice.

	bioluminescent intensity (×10^4^)		
group	mouse no. 1	mouse no. 2	mouse no. 3
control	7.33	2.08	7.12
early-stage tumour	69.2	31.6	37.1
late-stage tumour	434	191	305

**Figure 3.**
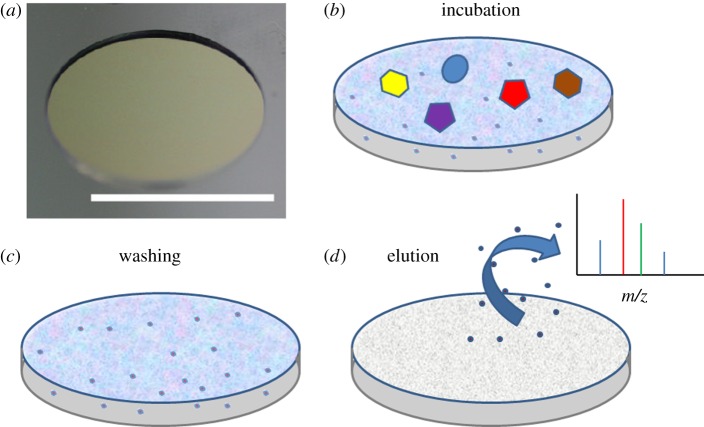
Schematic of enrichment of LMW proteins and peptides on a nanochip. (*a*) A unit on a nanoporous silica chip for sample processing. (*b*) After sample spotting on the surface, LMW proteins and peptides were trapped in the pores, while the larger species remained outside the pores. (*c*) These were removed during the washing steps. (*d*) Enriched fractions were eluted and analysed by MALDI. (Online version in colour.)

**Figure 4.**
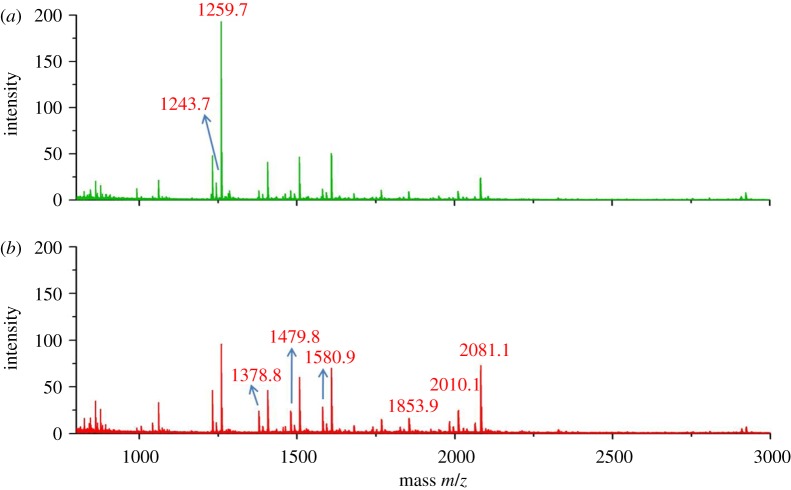
The average MALDI-TOF mass spectrum of mice serum between (*a*) normal control and (*b*) BCLM groups. Mass spectra obtained after serum samples were processed on F-127 nanochip. The 1243.7 and 1259.7 Da peaks were downregulated in (*a*), and the 1378.8, 1479.8, 1580.9, 1853.9, 2010.1 and 2081.1 Da peaks were upregulated in (*b*). (Online version in colour.)

The schematic for serum fractionation using the mesoporous silica (MPS) thin films is shown in figure 3. After 30 min of pretreatment with 5 per cent ACN and 0.01 per cent TFA, 7 μl of the serum sample was added to each well on the MPS thin film surface and incubated for 30 min in a humidified box. The LMW proteins and peptides were loaded into the nanopores, while large proteins, such as albumin, immunoglobulin G, etc., were removed by washing the nanochip surface several times with deionized water. The enriched LMW proteins and peptides were eluted with 5 μl of 50 per cent ACN and 0.1 per cent TFA. These eluted samples were analysed with MALDI-TOF MS to obtain profiles of the LMW proteins and peptides in mouse serum from all three groups. The peptides in mouse serum were enriched and could be detected by MALDI-TOF MS. Furthermore, the average spectrum appeared widely different in the low-mass range (800–3000 Da) between the control and the tumour groups based on their MALDI-TOF MS spectra (figure 4).

**Figure 5.**
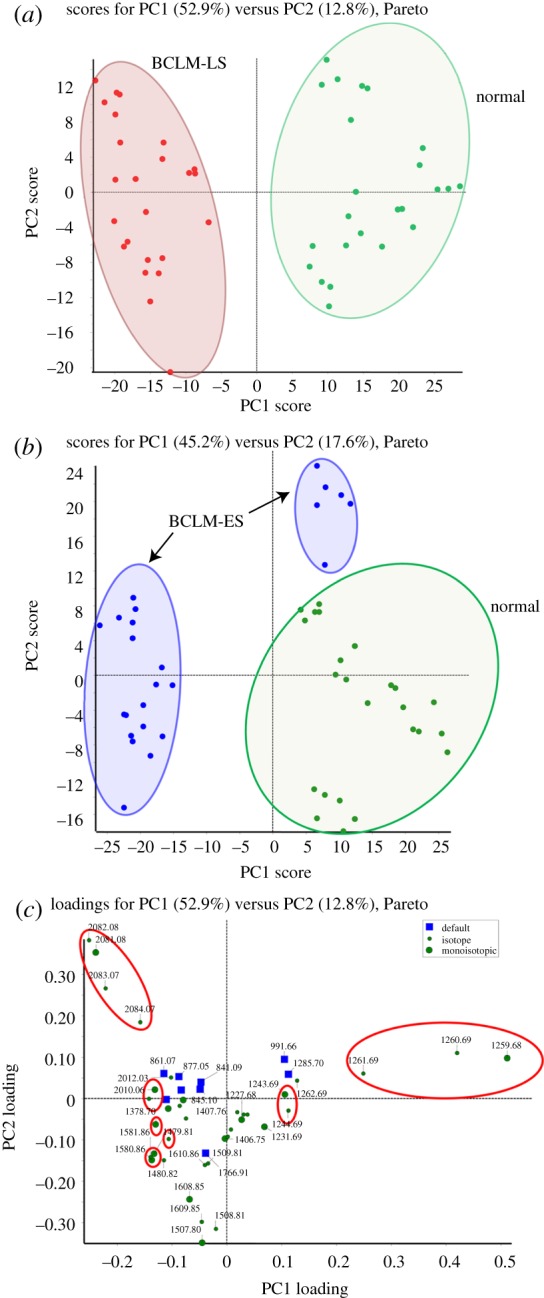
PCA analysis of control, BCLM-LS and BCLM-ES groups. PC1 and PC2 scores obtained through PCA analysis of the replicated MALDI-TOF MS datasets in three groups. (*a*) The scores plot of PC1/PC2 between the BCLM-LS and control groups. (*b*) The scores plot of PC1/PC2 between the BCLM-ES and control groups. (*c*) The loadings plot specific for PC1/PC2 between the BCLM-LS and control groups. Highlighted ion clusters represent specific peptides for classification within these two groups. (Online version in colour.)

For statistical analysis of the profiles for LMW proteins and peptides acquired after processing on nanochips, we applied PCA using MarkerView. PCA is a statistical procedure commonly used for reducing dimensional variables, while still maintaining most of the original series [28]. For PCA analysis, the first principal component (PC1) and the second principal component (PC2) determine the first and the second greatest amounts of variance. After PCA processing using MarkerView, the principal components (PCs) were calculated and plotted for each sample score PC1 and PC2, as shown in figure 5*a*. In this study, for the BCLM-LS and control groups, PC1 yielded 52.9 per cent of the variance, and PC2 12.8 per cent. The score plot of PC1/PC2 indicates that the BCLM-LS group was completely separated from the control group based on their PC1 score. The control group (right-hand cluster, green symbols) received positive PC1 scores, while the BCLM-LS group (left-hand cluster, red symbols) attained negative scores. Therefore, PCA analysis suggests that potential exists for using the LMW protein and peptide profiles obtained after processing on a nanochip as a diagnostic tool for distinguishing between tumour and non-tumour groups. The results for early-stage (BCLM-ES) samples compared with the control group were also analysed with PCA procedures, as shown in figure 5*b*. Slightly different from the profiling of BCLM-LS, the BCLM-ES samples (left-hand and upper clusters, blue symbols) received both positive and negative PC1 scores, but could still be separated from the control group based on PC score, indicating that nanoporous chip-based technology provides a potential pathway for biomarker discovery for early-stage metastatic breast cancer. The loadings plot in figure 5*c* demonstrates the PC1 and PC2 loadings for each variable, indicating how each variable contributes to the PCs. In other words, the PC loading plots reveal peptides that can be used to specifically distinguish between different groups. The peptides with the larger PC loadings are the more important variable and thus have more potential as diagnostic biomarkers for classifying tumour and non-tumour groups. The peptide points with the higher loadings are highlighted (red ellipses) in figure 5*c*.

**Figure 6.**
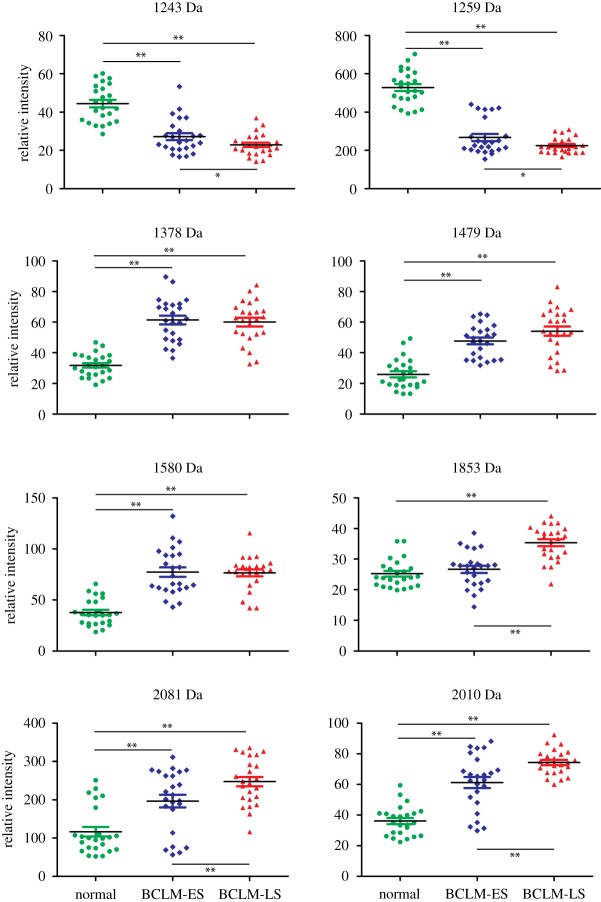
Different expressed biomarker candidates between the normal control group and the breast cancer lung metastasis groups in mice serum. Eight biomarker candidates were detected. Significance: *p<0.05; ^**^p<0.01. (Online version in colour.)

To examine the specific peptides revealed by the loadings plot, a *t*-test analysis was performed using MarkerView. The *t*-test was carried out by comparing each group to the remaining two groups, to determine how the variable distinguished these three sets. The peptides highlighted in the loadings plot showed low *p*-value between the control group and the BCLM-LS group, which indicated good agreement with PCA analysis. The vertical scatter plots of eight specific biomarkers shown in figure 6 exhibit the variable expressed level of the specific LMW proteins or peptides during the three different stages of breast cancer lung metastasis. Peptides at *m*/*z*=1243 and 1259 Da were significantly downregulated in both BCLM-ES and BCLM-LS groups at the early and late stage of BCLM in order to compare with the control group. The other six peptides, at *m*/*z*=1378, 1479, 1580, 1853, 2010 and 2081 Da, respectively, were significantly upregulated in BCLM-ES and BCLM-LS groups, except for the peptide at 1853 Da in the BCLM-ES group, which exhibited the same level as in our control group. The level of fragments at *m*/*z*=1243 and 1259 Da was significantly downregulated in the control group mice compared to both early-stage and late-stage tumour mice. The level of the five fragments at *m*/*z*=1378, 1479, 1580, 2010 and 2081 Da was significantly upregulated in both groups of mice with tumours.

It is interesting to note that the candidate biomarkers can be divided into three groups (figure 6). Group 1 fragments include 1243, 1259, 1378 and 1580; fragment 1853 is the only member in group 2; and group 3 fragments are 1479, 2081 and 2010. Changes in serum level of group 1 fragments could be detected in both BCLM-ES and BCLM-LS mice. These fragments can serve as biomarkers for detection of early-stage tumour metastasis, as elevated levels of the fragments could be detected in mouse serum as early as two weeks after tumour inoculation. A similar basal level of fragment 1853 could be detected in control mice and BCLM-ES mice, but the level is significantly elevated in BCLM-LS mice. This fragment is indicative of late-stage tumours. The level of group 3 fragments increased during tumour development, and reached a peak in BCLM-LS mice. Analysis of expression signatures based on the mass spectrum could thus be used to determine which stage of cancer development a patient is in. Application of biomarker analysis in the clinic could guide physicians to treat cancer patients better based on their disease progression. These expression signatures can also serve as biomarkers to monitor therapeutic efficacy for patients under drug treatment. For example, the trend of group 3 markers may very well be reversed if the tumour mice were treated with chemotherapy or targeted therapy drugs. More detailed studies in mass sequence are needed to validate the usefulness of these biomarkers.

## 4. Conclusion

Nanoporous silica chips are very efficient in capturing LMW proteins and peptides from the serum of mouse models of human breast cancers. Once isolated, small proteins and peptides in this study have unique levels of expression for different stages of BCLM. Further characterization studies of these proteins and peptides will identify even better the biomarkers that are indicative of cancer development. Thus, the nanotechnology-based silica chips provide a rapid, sensitive, reproducible and inexpensive platform that will lead to significant, personalized diagnosis and new therapies for the treatment of breast cancer.
